# Comparison of Fitness Cost, Stability, and Conjugation Frequencies of *tet*(X4)-Positive Plasmids in Chicken and Pig *Escherichia coli*

**DOI:** 10.3390/antibiotics11111657

**Published:** 2022-11-19

**Authors:** Ziyi Liu, Huiru Zhang, Xia Xiao, Yuan Liu, Ruichao Li, Zhiqiang Wang

**Affiliations:** 1 College of Veterinary Medicine, Yangzhou University, Yangzhou 225009, China; 2 Jiangsu Co-Innovation Center for Prevention and Control of Important Animal Infectious Diseases and Zoonoses, Yangzhou 225009, China; 3 Institute of Comparative Medicine, Yangzhou University, Yangzhou 225009, China

**Keywords:** fitness cost, *tet*(X4)-bearing plasmids, pig *E. coli*, chicken *E. coli*, plasmid persistence and dissemination, tetracyclines, plasmid conjugation

## Abstract

The large-scale epidemic of the *tet*(X4) gene in the livestock and poultry industry is threatening public health; however, there is still a lack of comparative studies on *tet*(X4)-bearing plasmids in chicken and pig *Escherichia coli*. To evaluate the prevalence trend of *tet*(X4)-bearing plasmids and the factors influencing their persistence in the livestock and poultry industry, we examined the fitness cost, stability under tetracyclines pressure, and conjugation frequencies at various temperatures of six *tet*(X4)-bearing plasmids in four representative pig *E. coli* isolates and chicken *E. coli* isolates. Compared with pig *E. coli*, the plasmid in chicken *E. coli* showed lower fitness cost, and stronger ability to promote bacterial biofilm formation and motility. Meanwhile, the presence of tetracycline may favor the stability of *tet*(X4)-bearing plasmids, which was more common in chicken *E. coli*. Furthermore, the optimal temperature for IncX1 *tet*(X4)-bearing plasmid conjugation was 42 °C, and its conjugation frequency in chicken *E. coli* was higher than that in pig *E. coli*, whereas the optimal temperature for IncFII *tet*(X4)-bearing plasmid conjugation was 37 °C and it performed better in pig *E. coli*, suggesting the predominant plasmid types circulating in chicken *E. coli* and pig *E. coli* may be distinct. Collectively, although *tet*(X4) currently appears to be more prevalent in pig *E. coli*, this is probably independent of the fitness cost caused by *tet*(X4)-plasmids. To curb the future spread of the *tet*(X4) gene, reduced tetracyclines usage and tailored interventions should be applied in different breeding industries.

## 1. Introduction

In 2019, the emergence of plasmid-encoded *tet*(X) genes conferring tigecycline resistance raised public concerns [1]; it seriously threatened the effectiveness of tigecycline. The spread of the *tet*(X4) gene is mainly mediated by different types of plasmids, such as IncX1, IncQ1, IncFIB, IncHI1, IncFII, and hybrid plasmids, among which the IncX1 plasmid is the dominant type [2]. According to a large-scale epidemiological investigation encompassing 4189 samples from animal farms and human specimens, the *tet*(X4)-positive strains were exclusively found in strains of animal origin, and *Escherichia coli* was the sole bacterial host [3]. *tet*(X4) has a high detection rate in animal breeding industry; however, the distribution of *tet*(X4) is biased in pig *E. coli* and chicken *E. coli*. In 240 samples from a study conducted by our group, 74 *tet*(X4)-positive *E. coli* (30.8%) isolates were isolated, demonstrating the widespread prevalence of *tet*(X4)-positive strains in pig slaughterhouses [4]. Another investigation into the frequency of *tet*(X4)-positive strains in pig farms identified 32 *tet*(X4)-positive strains (20.1%) among 159 samples as well [5]. *tet*(X4)-positive *E. coli*, in contrast, was relatively rare in the poultry breeding industry. A previous study only collected three *E. coli* strains from poultry chicken meat, wild bird, and the slaughterhouse wastewater in Pakistan, and these strains were found in a variety of phylogenetic clades [6]. Similarly, another study identified four *tet*(X4)-positive *E. coli* (8.9%) isolates in 45 chicken fecal samples [7]. Recently, a systematic retrospective study collected 613 *tet*(X4)-positive *E. coli* isolates from public databases, and found that pigs and their products were the most common vehicles (n = 162) for *tet*(X4) dissemination, followed by human (n = 122), chickens (n = 60), and the environment (n = 49) [8], suggesting a substantial difference in the prevalence of *tet*(X4) between chicken *E. coli* and pig *E. coli* [2]. Nevertheless, the underlying reason for this distribution difference remains unclear.

In order to explain the above phenomenon, we hypothesized that there may be three underlying reasons for the variation in *tet*(X4)-positive *E. coli* distribution. First, the genetic characteristics of pig *E. coli* and chicken *E. coli*, especially pathogenic *E. coli*, are different. Avian pathogenic *E. coli* (APEC) is the most common pathogenic *E. coli* in chickens, mainly belonging to O1, O2, and O78 serotypes [9], whereas Enterotoxigenic *E. coli* (ETEC) and Shiga-toxigenic *E. coli* (STEC) are important pathogens in swine breeding [10], and serogroups such as O8, O138, O139, O141, O147, O149, and O157 have been frequently reported in pig colibacillosis worldwide [11]. Therefore, the *tet*(X4) or *tet*(X4)-bearing plasmids may induce various degrees of fitness cost in pig and chicken *E. coli* [12]. Second, the tetracyclines class is the most widely used antibiotic in global pig and chicken production chain [13], which may encourage the persistence and evolution of tetracyclines resistance, but this process may be influenced by other factors, such as animal physiological characteristics and farm environment, thereby causing the distribution differences of *tet*(X4). Third, the different body temperatures of chickens and pigs may lead to a difference in the conjugation frequencies of *tet*(X4)-bearing plasmids. Therefore, we aim to comprehensively evaluate the fitness cost of *tet*(X4)-bearing plasmids on pig and chicken *E. coli*, and to investigate the effect of tetracyclines and temperatures on the stability and conjugation frequencies of *tet*(X4)-bearing plasmids, respectively, in these two types of bacteria.

## 2. Results

### 2.1. Fitness Cost of the Tet(X4)-Bearing Plasmids in Engineered Bacteria TOP10, Pig E. coli, and Chicken E. coli

**Figure 1:**
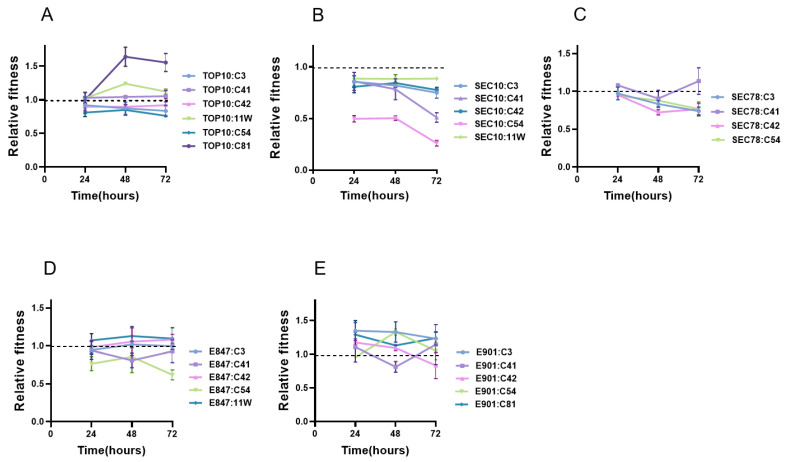
Relative fitness indices for 72 h of the transformants with their isogenic plasmid-free strains in this study. (**A**): Transformants of TOP10; (**B**): transformants of SEC10; (**C**): transformants of SEC78; (**D**): transformants of E847; (**E**): transformants of E901.

In this study, we attempted to transfer six *tet*(X4)-bearing plasmids of pig or chicken origin into five *E. coli* isolates, including the engineered bacteria TOP10, two pig *E. coli* isolates, and two chicken *E. coli* isolates, and successfully obtained 25 matching transformants (Table 1). In vitro competitive testing was used to evaluate the fitness cost of these plasmids in various hosts. The results showed that these plasmids imposed little or no fitness cost on TOP10, with the relative fitness of TOP10:C3, TOP10:C42, and TOP10:C54 being slightly below 1, whereas the carriage of plasmid C81 enhanced the competitive ability of TOP10. In terms of pig *E. coli* strains, all the plasmids that could be introduced into SEC10 had an obvious fitness cost, with plasmid C54 producing the highest fitness cost. Except for C41, all the plasmids exerted fitness cost in four transformants of SEC78, and their relative indices declined as the culture period increased. In contrast, these plasmids produced a lower fitness cost in chicken *E. coli*, since the relative indices of most plasmids were higher than 1. Compared with other host bacteria, strain E901 could be regarded as the ideal host to spread *tet*(X4)-bearing plasmids, since none of the plasmids impose an obvious fitness cost in E901 (Figure 1). These findings indicated that the fitness cost of these *tet*(X4)-bearing plasmids in chicken *E. coli* was generally lower than that in pig *E. coli*.

### 2.2. Biofilm Formation of the Tested Strains after Acquiring Various Tet(X4)-Bearing Plasmids

**Figure 2:**
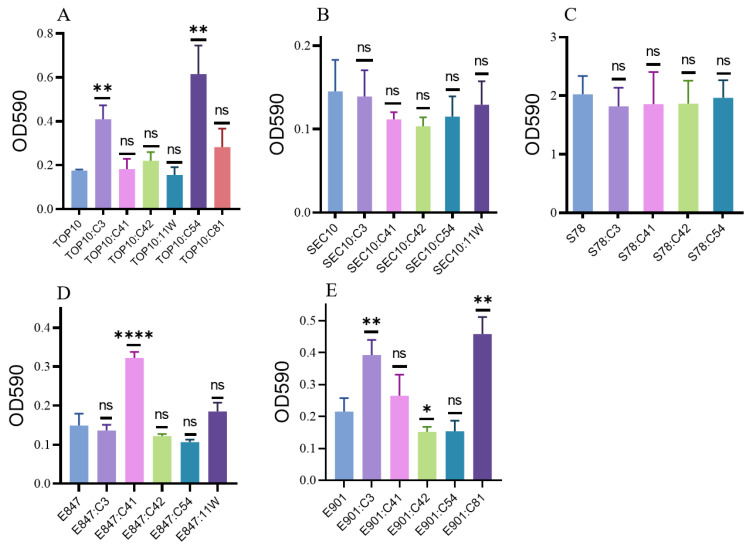
Biofilm formation ability of the transformants and their isogenic plasmid-free strains in this study. (**A**): Transformants of TOP10; (**B**): transformants of SEC10; (**C**): transformants of SEC78; (**D**): transformants of E847; (**E**): transformants of E901. Data are shown as mean ± SD. *p*-values were determined using the *t*-test (* *p* < 0.05, ** *p* < 0.01, **** *p* < 0.0001, ns, not significant).

The capacity of bacteria to produce biofilm is a crucial sign of their pathogenicity and resistance to antibiotics [14]. After obtaining several *tet*(X4)-bearing plasmids, the biofilm formation ability of these transformants was evaluated. Acquisition of plasmid C3 and C42 considerably increased the biofilm production of TOP10. Interestingly, none of the tested pig *E. coli* strains significantly altered the biofilm production. However, chicken *E. coli* was more conducive to the survival and transmission of *tet*(X4)-bearing plasmids, as the ability of several plasmids increased the biofilm production in chicken *E. coli*, including plasmid C41 in E847, plasmid C3 in E901, and plasmid C81 in E901 (Figure 2).

### 2.3. Swimming Motility of the Tested Strains after Acquiring Various Tet(X4)-Bearing Plasmids

**Figure 3:**
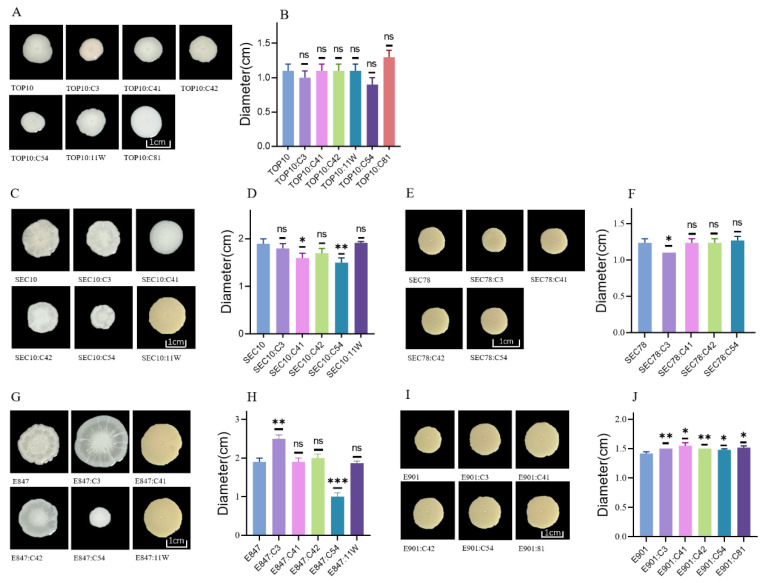
Swimming motility of the transformants and their corresponding isogenic plasmid-free strains. (**A**): Swimming motility of TOP10 and its transformants; (**B**): the mean diameters of TOP10 and its transformants; (**C**): swimming motility of SEC10 and its transformants; (**D**): the mean diameters of SEC10 and its transformants; (**E**): swimming motility of SEC78 and its transformants; (**F**): the mean diameters of SEC78 and its transformants; (**G**): swimming motility of E847and its transformants; (**H**): the mean diameters of E847 and its transformants; (**I**): swimming motility of E901 and its transformants; (**J**): the mean diameters of E901 and its transformants. Data are shown as mean ± SD. *p*-values were determined using the *t*-test (* *p* < 0.05, ** *p* < 0.01, *** *p* < 0.001, ns, not significant).

Bacterial swimming motility is related to the invasiveness and adhesion ability [15], and a pervious study suggested that the introduction of exogenous *tet*(X4)-bearing plasmids may have an impact on bacterial movement ability [16]. Therefore, we compared the changes of motility in these tested strains after acquiring *tet*(X4)-bearing plasmids. As results showed, TOP10’s swimming motility did not change significantly after receiving different *tet*(X4)-bearing plasmids, while pig *E. coli* strains with plasmid C3 in SEC78, C41 in SEC10, and C54 in SEC10 decreased swimming motility. In contrast, the swimming motility of E901 and E847 was improved by all plasmids and plasmid C3, respectively (Figure 3).

### 2.4. Effect of Tetracycline on the Stability of Tet(X4)-Bearing Plasmids

**Figure 4:**
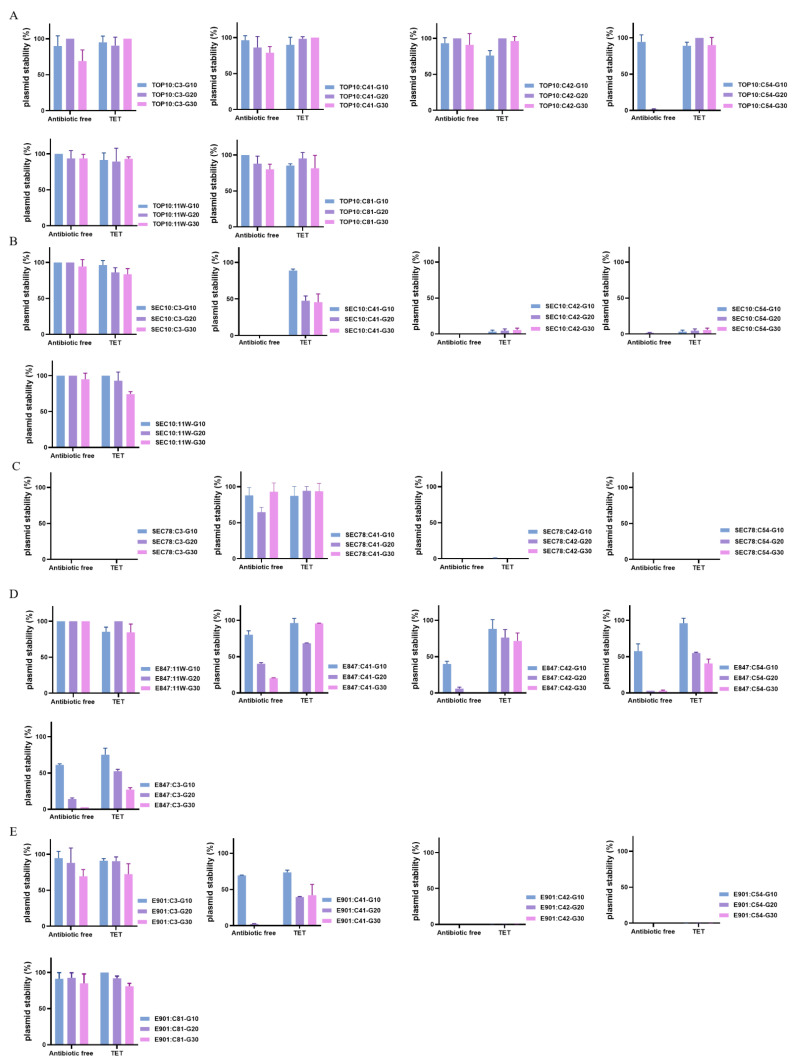
Stability of *tet*(X4)-bearing plasmids in the transformants of different generations. (**A**): Transformants of TOP10; (**B**): transformants of SEC10; (**C**): transformants of SEC78; (**D**): transformants of E847; (**E**): transformants of E901.

Although tigecycline is banned for use in animal husbandry, other tetracyclines, such as tetracycline, are commonly utilized. Thus, the stability of these *tet*(X4)-bearing plasmids was examined after 30 generations of serial passaging in tetracycline-containing and antibiotic-free broth to determine if the usage of tetracyclines could increase the survival rate of *tet*(X4)-bearing plasmids. Except plasmid C54, the remaining plasmids in TOP10 remained stable during serial passaging. Plasmid C54 was rapidly eliminated in antibiotic-free broth; however, the presence of tetracycline can effectively enhance the plasmid stability. In addition, several plasmids, such as C42 and C54, in pig *E. coli* strains were unstable in the presence or absence of tetracycline, but plasmid C41 in SEC10 showed a marked improvement in stability in the presence of tetracycline. For chicken *E. coli*, while plasmid 11W in E847 and plasmid C3 or plasmid C81 in E901 could steadfastly persist in their host bacteria, the stability of several plasmids, including C3, C41, C42, and C54 in E847 and C41 in E901, showed varying degrees of elevation in the presence of tetracycline (Figure 4). These results clearly showed that the residue of tetracycline could increase risk of *tet*(X4)-bearing plasmid persistence, which will facilitate further plasmid evolution and dissemination.

### 2.5. Effect of Temperature on Conjugation Frequencies of the Tet(X4)-Bearing Plasmids

**Figure 5:**
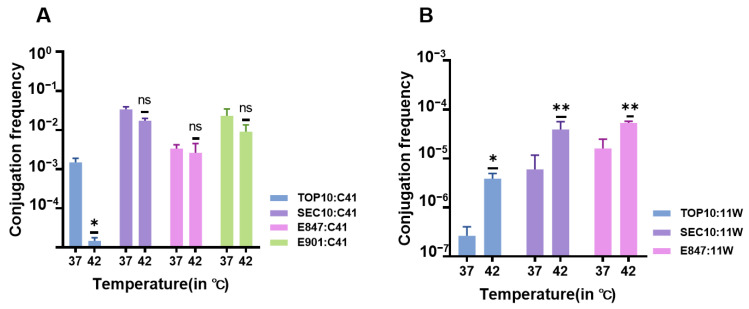
Conjugation frequencies of *tet*(X4)-bearing plasmids at 37 °C and 42 °C. (**A**): Conjugation frequencies of plasmid C41 in TOP10, SEC10, E847, and E901; (**B**): conjugation frequencies of plasmid 11W in TOP10, SEC10, and E847. *p*-values were determined using the *t*-test (* *p* < 0.05, ** *p* < 0.01, ns, not significant).

The normal body temperature of pigs ranges from 38–39 °C, and that of chickens is approximately 42 °C. Hence, we investigated the conjugation frequencies of *tet*(X4)-bearing plasmids at 37 °C and 42 °C to mimic the effect of different body temperatures on the plasmid conjugation frequency. Unfortunately, only IncFII plasmid C41 and IncX1 plasmid 11W were conjugative. At 37 °C, plasmid C41’s conjugation frequency was much greater than that at 42 °C in TOP10, which climbed by approximately two orders of magnitude. Similar tendencies were observed in other host strains, but it did not constitute significant differences, suggesting that 37 °C may be more favorable for IncFII *tet*(X4)-bearing plasmid conjugation. Furthermore, the conjugation frequency of the IncFII *tet*(X4)-bearing plasmid showed superior performance in pig *E. coli*. On the contrary, the conjugation frequency of the IncX1 plasmid was greater at 42 °C than that at 37 °C, and it seemed to spread more easily in chicken *E. coli* (Figure 5).

## 3. Discussion

Plasmids usually impose a fitness cost on host bacteria, which manifests as a decreased bacterial growth, diminished competitiveness, and decreased virulence [17]. The results of the three bacterial fitness indicators (competitiveness, biofilm formation ability, and motility) together showed that the *tet*(X4)-bearing plasmids generally caused a lower fitness cost in chicken *E. coli* than in pig *E. coli*, which may not support the prevailing view that *tet*(X4) is more prevalent in pig *E. coli*. Therefore, we believe that the distribution difference may be jointly affected by multiple factors. The previous study suggested that the emergence of the *tet*(X4) gene in farm animals in China was a recent event [18], because a retrospective study demonstrated that the *tet*(X4) gene was identified in pig *E. coli* isolated after 2016 during 2008–2018 [19]. Given that both pig *E. coli* and chicken *E. coli* in this study were isolated before 2016, it is necessary to expand the range of tested host bacteria to get a deeper understanding in the future research. Biofilm formation ability and swimming motility serve as important indicators for bacterial adaptation and virulence [16], among which biofilm can assist bacteria to withstand environmental pressure [20] and swimming motility is associated with bacterial invasiveness and adhesion [15]. In this study, chicken *E. coli* generally displayed better biofilm formation ability and motility, implying chicken *E. coli* may be more threating after obtaining *tet*(X4)-plasmids. Furthermore, we speculate that the distribution difference may be attributed to the intestinal environment of the two animals. The evidence was that a previous study analyzed the diversity of antibiotic resistance genes (ARGs) in intestinal microbiota metagenomes of pigs, human, and chickens, and found that the detection rate of the *tet*(X) gene in pigs was higher than that in chickens [21], which implied that the prevalence of the *tet*(X) gene was probably affected by the intestinal environment of different animals. Since chicken gut temperature is higher than pigs, and a recent study has shown that high temperature can effectively degrade tetracycline residues in chicken manure [22], we hypothesized that gut temperature may be one of the factors affecting distribution difference of *tet*(X4) in the two animals.

Compared with other antibiotics, tetracyclines are widely used in many countries because they are relatively cheap and cost-effective [13,23]. Nevertheless, our results revealed that the presence of tetracyclines could help improve the stability of *tet*(X4)-bearing plasmids, especially in chicken *E. coli*. According to an earlier study, tetracyclines are still used for the prevention and treatment of diseases and promotion of growth in animals in many countries [24]. More seriously, more than 75% of tetracyclines are released into the environment through animal urine and feces [25]. Because all the host bacteria in this study were resistant to tetracycline, the *tet*(X4)-bearing plasmid was not essential for bacterial survival under tetracycline pressure. Combined with our results, we believed that these adverse factors may further aggravate the dissemination of *tet*(X4). According to the successful precedent of a sharp decrease in the abundance of the *mcr-1* gene in animals and humans after colistin was banned as a growth promoter in the animal industry [26], in order to curb the prevalence of animal-derived *tet*(X4) gene, rational or reduced use for tetracyclines is crucial.

Currently, conjugation of several plasmids has been demonstrated to be temperature sensitive. For example, the optimal temperature for IncHI plasmids is between 22 °C and 30 °C [27]; such conjugative characteristics may be an important abiotic factor affecting its distribution [28]. Therefore, we evaluated the effect of temperature on the conjugation frequencies of different *tet*(X4)-bearing plasmids as well. The IncX1 plasmid exhibited a better transferability at 42 °C in chicken *E. coli*, which may be because the plasmid was originally derived from chicken *E. coli*. It has been reported that the dominant plasmids from chicken and mammals were distinct. For instance, a study found that IncK2 plasmids have a higher conjugation frequency at 42 °C compared with IncK1 plasmids, and the increased expression of a regulator of heat-shock protein in *E. coli* with IncK2 at 42 °C may explain why IncK2 plasmids were predominantly found in chicken isolates [29]. Moreover, it has been found that the conjugation of *tet*(O)-carrying plasmids in *Campylobacter* is thermoregulated, making it occur preferentially in birds [30]. Although no relevant studies have shown that IncX1 plasmids are the dominant type in poultry, our results demonstrated the transmission potential of the plasmid in chicken *E. coli*, which warrants further investigation.

There are still some limitations in this study. First, the number of the selected *E. coli* of pig and chicken origin are relatively few, and they have been isolated for a long time. Second, to confirm our hypothesis, further epidemiological survey data are required. For example, whether the detection rate of *tet*(X4) in chickens will increase and IncX1 plasmids will become the major plasmids in the dissemination of *tet*(X4) in chicken farms.

## 4. Materials and Methods

### 4.1. Bacterial Strains and Plasmids

The pig *E. coli* SEC10 and SEC78 were isolated from diseased pigs, and chicken *E. coli* E847 and E901 were isolated from diseased chickens. Among them, E901 and E847 belong to ST1196 and ST155, which are representative ST types of APEC [31,32], whereas SEC10 belongs to ST88, a high-risk clone that was usually isolated from diseased pigs [33,34]. Unfortunately, the ST type of SEC78 has not been determined. The detail information of these strains was listed in Supplementary Material Table S1, and their antimicrobial resistance profiles were listed in Supplementary Material Table S2. In addition, we selected six *tet*(X4)-bearing plasmids isolated from chicken feces or pig slaughterhouse in our previous study [4,35] for further study. These plasmids belong to IncX1, IncFII, IncA/C2, IncFIA/IncHI1A/IncHI1B, IncFIA/IncHI1A/IncHI1B/IncX1, and IncX1, respectively (Supplementary Material Table S3).

### 4.2. Construction of Transformants

To evaluate the fitness cost in pig or chicken *E. coli*, these plasmids and strains were pairwise combined, and the plasmids were transferred into host bacteria by electroporation. The transformants were screened on LB agar plates (Haibo Biotechnology Co., Ltd., Qingdao, China) containing tigecycline (4 μg/mL) and confirmed by PCR targeting *tet*(X4) gene. The primers for *tet*(X4) were forward: 5′-CCGATATTCATCATCCAGAGG and reverse: 5′-CCGATATTCATCATCCAGAGG as previously mentioned [1]. Genomic DNA of the *tet*(X4)-positive strain RF45-1 was used as the positive control [4].

### 4.3. Pairwise Competition Assay

According to the previous study [36], overnight cultures of transformants and their isogenic plasmid-free strains were diluted to a 0.5 McFarland standard and mixed at the ratio of 1:1 in 5 mL LB broth (Haibo Biotechnology Co., Ltd., Qingdao, China). The mixtures were incubated at 37 °C for 72 h with shaking. Every 24 h, 5 μL of mixtures was reinoculated into a fresh 5 mL of LB broth. Meanwhile, an aliquot of 50 μL mixtures was taken out and then was tenfold serially diluted. The diluents were plated on LB agar plates with or without tigecycline (4 μg/mL) to calculate the number of cells for each strain. The relative fitness was calculated as follows: w = ln (NRt/NR0)/ln (NSt/NS0). NR: number of resistant clones; NS: number of susceptible clones, with value below one indicating the existence of fitness cost.

### 4.4. Biofilm Formation Assay

Biofilm formation was quantified using crystal violet staining [16]. Briefly, overnight cultures were adjusted to a cell density equivalent to a 0.5 McFarland standard. 200 μL of bacteria suspension was added to a 96-well plate and incubated at 37 °C for 48 h. After incubation, biofilms were attached to the surface of the wells. Cultures were discarded carefully and wells were washed twice with 200 μL PBS. The biofilms were fixed in methanol for 10 min. The methanol was removed, and the biofilms were stained with 0.1% crystal violet solution for 10 min and rinsed with PBS until colorless. Subsequently, the biofilms were dissolved in 100 μL of 30% formic acid for 30 min, and biofilm formation was quantified by measuring the absorbance at OD 590 nm.

### 4.5. Motility Test

The movement ability of bacteria was determined by the method of semisolid medium as previously mentioned [37]. Overnight cultures were adjusted to a 0.5 McFarland and were diluted 100-fold with fresh LB broth. Then, 0.3% LB semisolid medium (3 g/L) agar plates (Haibo Biotechnology Co., Ltd., Qingdao, China) were prepared and 2 μL of bacterial solution was plated on the center of each plate. After the plates were incubated at 37 °C for 48 h, the diameter of each colony was measured by a ruler. Experiments were conducted with three biological replicates.

### 4.6. Stability of Tet(X4)-Bearing Plasmids during Serial Passaging

To evaluate whether the presence of tetracyclines could favor the stability of *tet*(X4)-bearing plasmids, the transformants were propagated by serial transfer for 15 days in tetracycline-containing (16 μg/mL), and antibiotic-free LB broth at 37 °C, respectively. Every 12 h, 5 μL of each culture was transferred into 5 mL fresh corresponding LB broth, and each passaging was defined as one generation. At the end of passaging, 100 μL of bacterial solution of the 10th, 20th, and 30th generations was serial tenfold diluted and plated on LB agar with or without tigecycline to determine the number of plasmid-containing cells in the population. Experiments were conducted with three biological replicates.

### 4.7. Conjugation Frequencies of Tet(X4)-Bearing Plasmids at 37 °C and 42 °C

To compare the conjugation frequencies of *tet*(X4)-bearing plasmids at different temperatures, measurements of conjugation frequency at 37 °C and 42 °C were performed. Briefly, the transformants served as the donor strain and *E. coli* C600 (resistant to rifampin) was regarded as the recipient strain. Cultures of donor and recipient strain with a density of 0.5 McFarland were mixed at a ratio of 1:4. Subsequently, 100 μL of the mixtures was applied onto a sterile filtration membrane. The membrane was incubated on a LB agar plate without antibiotic at 37 °C and 42 °C for 12 h, respectively. The bacteria on the membrane were collected and serial tenfold diluted, and then were plated on the LB agar plate containing 200 mg/L rifampin or 200 mg/L rifampin and 4 mg/L tigecycline. Conjugation frequencies were calculated by the number of transconjugants per recipient cell.

### 4.8. Statistical Analyses

GraphPad Prism 8.3.2 was used to compare the data of relative fitness, biofilm for mation ability, plasmid stability, and conjugation frequency. Significant differences were assessed using the *t*-test, with *p* < 0.05 considered as statistically significant. 

## 5. Conclusions

In conclusion, our data indicated that the distribution difference of *tet*(X4) in pig *E. coli* and chicken *E. coli* may be independent of the fitness cost of *tet*(X4)-bearing plasmids. The irrational use of tetracyclines as growth promoters and tetracycline residue may aggravate the persistence of *tet*(X4)-bearing plasmids. Moreover, we predicted that the dominant *tet*(X4)-bearing plasmid was different in pigs and chickens, resulting in the diversity of the distribution of these plasmids between the two animals. Further studies need to expand the sample size, including host bacteria and *tet*(X4)-bearing plasmids, to get a deep insight into the differences in the distribution of *tet*(X4)-bearing plasmids between pigs and chickens.

## Figures and Tables

**Table 1:** Information of the selected plasmids and host bacteria. The first row of the table represents the six plasmids, and the first column of the table represents the five host bacteria. The six plasmids were electrotransformed into the five host bacteria respectively, and the successful transformants were named as “host bacteria:plasmid”.

Strains	C3 (Pig)	C41 (Pig)	C42 (Pig)	C54 (Pig)	C81 (Pig)	11W (Chicken)
TOP10	TOP10:C3	TOP10:C41	TOP10:C42	TOP10:C54	TOP10:C81	TOP10:11W
SEC10 (pig)	SEC10:C3	SEC10:C41	SEC10:C42	SEC10:C54	F	SEC10:11W
SEC78 (pig)	SEC78:C3	SEC78:C41	SEC78:C42	SEC78:C54	F	F
E847 (chicken)	E847:C3	E847:C41	E847:C42	E847:C54	F	E847:11W
E901 (chicken)	E901:C3	E901:C41	E901:C42	E901:C54	E901:C81	F

F: failed electroporation.

## Data Availability

Not applicable.

## Supplementary Materials

The following supporting information can be downloaded at: https://www.mdpi.com/article/10.3390/antibiotics11111657/s1, Table S1: Basic information of pig or chicken E. coli strains involved in this study; Table S2: MICs (mg/L) of four selected E. coli strains; Table S3: Basic information of *tet*(X4)-bearing plasmids in this study.
