# Supplementary material for: Comparison of Fitness Cost, Stability, and Conjugation Frequencies of tet(X4)-Positive Plasmids in Chicken and Pig Escherichia coli
Source: Antibiotics (Basel). 2022 Nov 19;11(11):1657. doi: 10.3390/antibiotics11111657 (PMC9686944; doi:10.3390/antibiotics11111657)
Supplement: Supplementary file 1 [file antibiotics-11-01657-s001.zip › antibiotics-2021017-supplementary.pdf]

Supplementary Materials

Table S1. Basic information of pig or chicken *E. coli* strains involved in this study.

	ST type	Isolation position	Isolation time	Serotype	FimH type
SEC10	ST88	Jinan, China	January 25, 2005	O8:H9	fimH39
SEC78	-	Rizhao, China	January 26, 2005	-	-
E847	ST155	Gaoyou, China	April 16, 2002	O8:H51	fimH32
E901	ST1196	Yangzhou, China	April 10, 2011	O173:H28	fimH31

Table S2. MICs (mg/L) of four selected *E. coli* strains.

	MIC (μg/mL)										
	**TIG**	**KAN**	**COL**	**AMP**	**ENR**	**CTE**	**FFC**	**MEM**	**OXY**	**CFF**	**TET**
SEC10	≤0.25	8	≤0.25	>128	16	64	4	≤0.25	>128	≤0.25	128
SEC78	≤0.25	>64	≤0.25	>128	8	32	32	≤0.25	128	≤0.25	128
E847	≤0.25	8	≤0.25	>128	≤0.5	64	4	≤0.25	>128	0.5	128
E901	≤0.25	>64	≤0.25	>128	32	32	8	≤0.25	128	128	64

TIG Tigecycline, KAN Kanamycin, COL Colistin, AMP Ampicillin, ENR Enrofloxacin, CTE Chlorotetracycline, FFC Florfenicol, MEM Meropenem, OXY Oxytetracycline, CFF Ceftiofur, TET Tetracycline.

Table S3. Basic information of *tet*(X4)-bearing plasmids in this study.

	Names in original study	Names in this study	Size	Replicon type	Reference
Pig slaughterhouse	pRF14-1_50k_tetX	C3	50518	IncX1	[4]
	pRF173-2_87k_tetX	C42	87445	IncA/C2	[4]
	pRF2-1_117k_tetX	C41	117635	IncFⅡ	[4]
	pRS3-2_194k_tetX_flye	C81	194164	IncFIA(HI1), IncHI1A, IncHI1B(R27)	[4]
	pRW7-1_235k_tetX	C54	235947	IncFIA(HI1), IncHI1A, IncHI1B(R27), IncX1	[4]
Chicken feces	pHNCF11W_tetX	11W	57105	IncX1	[18]
